# Conducting grounded theories in midwifery research: Practical insights^☆^

**DOI:** 10.1016/j.mex.2026.104047

**Published:** 2026-07-14

**Authors:** Martina Barbieri, Fiona Timmins, Milko Zanini, Loredana Sasso, Annamaria Bagnasco, Gianluca Catania

**Affiliations:** a Department of Health Sciences, University of Genoa, Via A. Pastore, 1, 16132 Genoa, Italy; b UCD School of Nursing, Midwifery and Health Systems, UCD College of Health Sciences, Belfield Dublin 4, Ireland

**Keywords:** Grounded theory, Midwifery, Methodology

## Abstract

This article offers a practice-oriented reflection on conducting Grounded Theory (GT) in midwifery research. Building on a constructivist GT study on Italian midwifery care, it does not restate standard GT procedures; instead, it focuses on the methodological decision points that most strongly shaped analytic quality, coherence, and feasibility. Rather than providing a generic overview, it traces the concrete steps, trade-offs, and adaptations involved in implementing GT in a real world, applied setting. The paper discusses the importance of explicit paradigmatic positioning, the central interpretive role of the researcher, and the need to preserve continuity between data collection and analysis. It also examines practical issues that are often underreported, including the organisation of episodes, early and ongoing memoing, data management, the limits of automation, and the time-intensive nature of analytic consolidation. By making these aspects explicit, the article aims to support researchers in planning and conducting GT studies with greater methodological awareness and rigour, especially in applied healthcare contexts. In doing so, it offers a transferable methodological template for designing and reporting constructivist GT studies in midwifery and related fields.

## Graphical abstract

**Figure:**
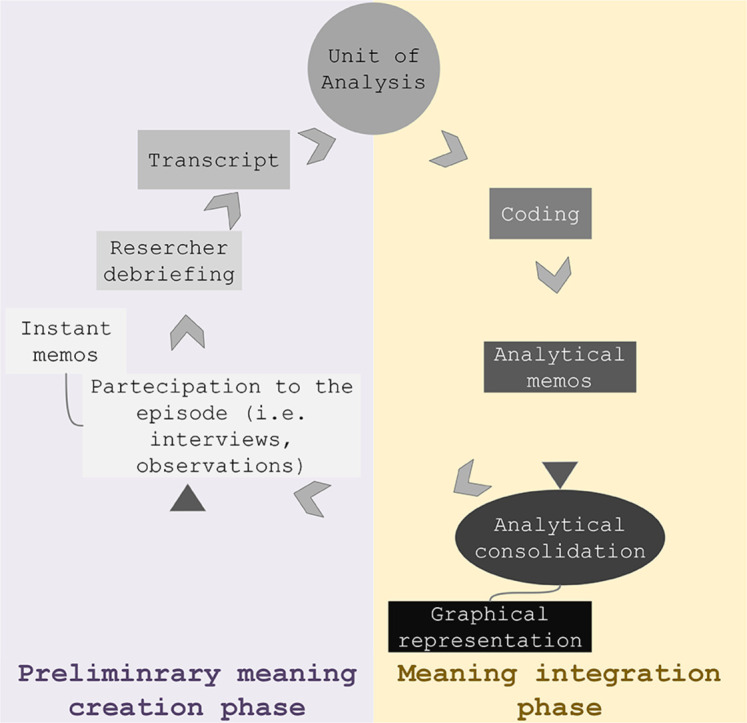

**Specifications table**

**Table:** 

**Subject area**	Medicine and Dentistry
**More specific subject area**	Midwifery
**Name of your methodology**	Grounded Theory
**Name and reference of original methodology**	Glaser, B., & Strauss, A. (1967). *The Discovery of Grounded Theory: Strategies for Qualitative Research*. Mill Valley, CA: Sociology Press. Corbin, J., & Strauss, A. (2015). *Basics of Qualitative Research (3rd ed.): Techniques and Procedures for Developing Grounded Theory*. SAGE Publications, Inc. https://doi.org/10.4135/9781452230153 Charmaz, K. (2006). Constructing Grounded Theory: A Practical Guide Through Qualitative Analysis. In *Introducing Qualitative Methods* (Vol. 1).
**Resource availability**	None

## Background

Midwifery research has a relatively short history and was initially shaped by medicine (Newnham & Rothman, 2022). Some of this influence is still visible today. Midwifery phenomena are still sometimes interpreted through a predominantly medical lens, and quantitative methods may be privileged as if they hold greater intrinsic value. These assumptions can sit in tension with the philosophical foundations of midwifery and with the complexity of the phenomena under study [1].

In response, midwifery researchers have increasingly engaged with methodological approaches better suited to exploring, interpreting, and explaining complex care practices and experiences [1]. Awareness of these issues has grown over time, supported in part by the increasing presence of PhD-prepared midwives and midwives with formal research training in academic settings [2].

This development is contributing to a broader shift in healthcare research. Midwives are entering research with stronger methodological preparation and greater confidence in qualitative inquiry. This is not only important for the development of the profession; it is also central to research quality [3]. As expectations for qualitative evidence in healthcare continue to rise, researchers must be able to justify, enact, and report methodological choices clearly and consistently. These capacities are essential for producing findings that are robust, credible, and professionally meaningful [4]. In this sense, methodological awareness is not an abstract academic concern but a practical requirement for high-quality research across designs, including Grounded Theory (GT) [3].

At the same time, GT includes multiple traditions, analytic emphases, and reporting expectations [5]. Methodological guidance is extensive, with three main approaches widely recognised in the literature [[6], [7], [8]]. Rigorous GT therefore requires more than adopting the label. It requires researchers to select a coherent methodological position, apply it consistently, and make that position explicit in both the conduct and reporting of the study [9].

However, in applied healthcare research, methodological principles are not always easy to translate into practical decisions for study design, analysis, and reporting [10]. This translation problem is the specific gap addressed by the present paper. The issue is not simply that qualitative research requires further justification within midwifery, but that researchers using GT must often make situated decisions that are only partially anticipated by standard methodological texts: how to align philosophical position and analytic practice, how to engage with existing literature without closing down theoretical sensitivity, how to move from descriptive coding to conceptual categories, how to judge theoretical sufficiency, and how to report an iterative analytic process transparently within applied healthcare constraints [11]. Drawing on experience gained through conducting a GT study in midwifery care, this paper is presented as a practice-oriented complement to the foundational GT literature rather than a substitute for it. In fact, we recognized that foundational texts, methodological overviews, and reporting guidelines provide essential guidance, but they cannot fully show how these decisions are negotiated within a specific empirical project, professional field, and healthcare context. It is assumed that readers are already familiar with the core GT traditions, concepts, and terminology. These are therefore not restated in detail, as they have already been clearly addressed in comparative methodological work [5,12,13]. Instead, the aim of this paper is to reflect on the methodological decision points that most strongly shaped the analytic process and the quality of the final account. The contribution is therefore deliberately focused: rather than offering another overview of GT or another general defence of qualitative inquiry, the paper examines the practical junctures at which methodological coherence can be strengthened or weakened when GT is used in applied midwifery research. By focusing on these practical and conceptual junctures, the paper seeks to offer useful reflections for both researchers with prior GT experience and those approaching GT for the first time.

## Methodology details

### Fundamental considerations

A first practical step in qualitative research is the development of a sound and relevant methodological background. This does not mean that only experts can conduct qualitative research. Rather, it points to the need for genuine engagement with the intellectual roots of the approach being adopted. Qualitative research includes a wide range of traditions, practices, and methodological orientations. At the same time, novice researchers can conduct high-quality qualitative studies, and doing so may itself be an important route to developing expertise [9].

For this reason, planning a qualitative study requires more than selecting interviews or focus groups as data collection methods. It requires engagement with ontology and epistemology, because these assumptions shape how research phenomena are understood and how knowledge is generated [14]. These dimensions underpin all research activity and reflect the philosophical position that informs methodological choices throughout the study [15].

Ontology concerns the nature of the reality under investigation, whereas epistemology concerns the relationship between researchers and that reality. Together, these assumptions inform methodology, that is, the practical way in which research is designed and conducted. They also contribute to the formation of broader paradigms, which orient research questions and, consequently, all subsequent research activities (Guba & Lincoln, 1994). Table 1 offers a simplified synthesis of paradigmatic assumptions, adapted from widely cited accounts of research paradigms. Although paradigms are useful as schematic reference points, they rarely operate in practice as fixed or sharply bounded categories. Researchers’ positioning may be nuanced, overlapping, and subject to change over time [18].

**Table 1:** Main features of most mainstream paradigms.

Paradigm	Ontology (nature of reality)	Epistemology (knower–known relationship)	Methodology (typical logic & methods)	Notes / typical quality focus
**Positivism** (Guba & Lincoln, 1994)	Single, objective reality (naïve realism)	Objective, value-free; distance between researcher and object	Deductive testing; experiments, measurement, prediction/control	Validity, reliability, generalisability
**Post-positivism** (Guba & Lincoln, 1994)	Reality exists but can be known only imperfectly (critical realism)	Modified objectivity; theory-laden observation; critical testing/falsification	Deductive/confirmatory with safeguards; experiments/quasi-experiments; triangulation/critical multiplism	Rigour via bias control, transparency, inference strength
**Constructivism / Interpretivism** (Guba & Lincoln, 1994)	Multiple, socially constructed realities (relativism)	Transactional/subjective; meaning co-constructed	Inductive/interpretive; qualitative designs (e.g., interviews, ethnography); hermeneutic/dialectic analysis	Credibility, reflexivity, contextual depth, trustworthiness
**Critical theory** (Guba & Lincoln, 1994)	Historical realism: realities shaped by social, political, cultural, economic, gendered power	Value-mediated; knowledge linked to critique of domination	Dialogic/dialectic; critical inquiry; may use qualitative + participatory approaches	Emancipation/critique; authenticity and transformative intent
**Transformative paradigm** [16]	Reality understood through structures of inequality; attention to oppression and social positioning	Knowledge is inseparable from values/ethics; explicit social justice orientation	Often participatory/mixed; methods chosen to expose inequities and support change	Axiology is central; human rights/social justice drive design choices
**Participatory paradigm** [17]	Subjective–objective reality; reality is co-created in participation	Extended epistemology (experiential, presentational, propositional, practical knowing)	Cooperative/participatory inquiry; co-researchers; action and reflection cycles	Practical knowing, democratic participation, co-ownership of inquiry

Paradigms are often discussed in relation to individual researchers, but they also operate at a disciplinary level. Scholarly communities sustain epistemic norms that influence what counts as a legitimate research problem, an acceptable method, and credible evidence. In health research, for example, the twentieth century was strongly shaped by positivist approaches to knowledge production, with randomized controlled trials often treated as an emblematic design in modern medicine (Newnham & Rothman, 2022). By contrast, interpretive traditions, which are foundational to several social-science disciplines, foreground meaning, context, and the situated nature of human action [19]. Applied healthcare disciplines, including midwifery, have therefore developed within a more plural methodological landscape, in which positivist/post-positivist, interpretive, and critical traditions are actively negotiated to produce knowledge that is both theoretically coherent and relevant to practice [4]. Making these disciplinary orientations explicit is especially important in interdisciplinary healthcare research, because paradigmatic assumptions may otherwise remain implicit while still shaping study design, analytic decisions, and the later interpretation and use of findings.

These issues require careful consideration before any research activity begins because paradigmatic orientation can function as a compass. It supports coherence between the research question, study aims, and the methodological choices that follow. Awareness of one’s paradigmatic positioning, of the plurality of paradigms, and of their implications for inquiry strengthens methodological reflexivity and increases the likelihood of producing high-quality and meaningful studies. It also helps researchers recognise the value of paradigms other than their own, thereby broadening their capacity to understand and appropriately assess alternative methods and findings [19].

This awareness also matters when research is read and evaluated. In this sense, the positionality section should not be treated as a merely formal component of the methods section. Rather, it provides important information about paradigmatic orientation and about the disciplinary, educational, and personal background that may shape the researcher’s role in the study. This is particularly important in qualitative research, where the researcher is the primary interpretive instrument through which the study is conducted [20].

Paradigmatic awareness, however, does not only concern how researchers position themselves; it also concerns whether the qualitative methods they use remain coherent with that positioning throughout the study. These ontological and epistemological considerations also clarify the distinction between what has been described as “small q” qualitative research and “Big Q” qualitative research. Big Q qualitative research refers to the use of qualitative methods within a qualitative paradigm, where knowledge is understood as situated and interpretive, and where meaning, context, and reflexivity are central to the production of findings. By contrast, small q qualitative research refers to the use of qualitative techniques, such as interviews or coding, without necessarily being grounded in a qualitative paradigm, often within realist/(post)positivist frameworks or as part of mixed-methods and instrument-development pipelines [21]. These are not merely stylistic differences. They involve different assumptions about what counts as evidence and therefore imply different quality criteria and reporting expectations. Without explicit awareness of this distinction, studies may unintentionally combine incompatible assumptions and produce what has been described as “confused q” qualitative research [22].

For these reasons, and in order to avoid the kind of "confused q" outcome described above, the GT study reflected on in this paper was deliberately approached as Big Q qualitative research rather than as the technical use of qualitative data-collection and coding procedures. This meant that methodological coherence was considered across the full research process: the formulation of the research question, sampling, interviewing, memo-writing, coding, category development, and reporting were all treated as shaped by qualitative assumptions about meaning, context, reflexivity, and situated knowledge. The study was located primarily within constructivist GT because participants’ accounts were understood as situated interpretations rather than neutral descriptions of a single reality, and because the analytic account was developed through interaction between participants’ meanings, the researcher’s interpretive work, and the wider empirical context (Charmaz, 2021). At the same time, the broader project and the topic of inquiry required a critical orientation. Critical theory informed the analysis by directing attention beyond individual experience to the institutional, professional, and policy conditions in which those experiences were produced. Analytically, this meant asking how power, professional hierarchy, dominant forms of evidence, and assumptions about legitimate maternity care shaped what participants described as possible, difficult, or taken for granted. The emancipatory element of this positioning did not involve claiming direct transformation of practice, but rather making visible the structures, constraints, and forms of professional agency that might otherwise remain implicit.

### The unique position of the researcher

Against this background, attention can now turn to the analytic core of GT. The concept that most clearly captures this core is constant comparison, widely described as the most distinctive feature of GT [9]. Constant comparison shapes both data collection and analysis so deeply that these activities, although often separated for teaching purposes, operate in practice as part of one continuous, cyclical, and iterative process [8].

Within this process, the role of the researcher is central. Experience gained through conducting a GT suggested that constant comparison is sustained by the researcher’s ongoing proximity to the data. Any contact with the data can therefore be understood as analytic work. This includes the research encounter itself, formal coding, memo writing, and all intermediate moments in which meanings begin to take shape.

A partial exception may be identified in the first episode of data collection, whether this takes the form of an interview, participant observation, or another method. This first episode is distinctive because it represents the initial encounter with participants’ meanings and is therefore not yet informed by prior analytic development. Even so, analytic engagement already begins at this stage through attentiveness, interpretation, and openness to what is emerging in the field.

For this reason, it is useful to retrace the main activities of a GT study by emphasising the central role of the researcher in meaning-making throughout the process. Because GT is methodologically complex, the process is discussed here through selected moments that are analytically distinguishable, even though in practice they are deeply interconnected. This segmentation is offered only as an expository device and should not be interpreted as a linear representation of GT.

The first cluster of activities may be understood as a *preliminary meaning creation phase*. This phase begins during each research episode and continues immediately afterwards through forms of early analytic engagement that preserve proximity to the data. Table 2 details the activities encompassed in the preliminary meaning creation phase and their description.

**Table 2:** Activities of the preliminary meaning creation phase and their description.

Activity	Description
Participation to the episodes	Regardless of the method used, participation in each episode is a core moment in the research process because it is the point of direct contact between the researcher, participants, and the setting. At this stage, the researcher is closest to what may be called the “raw meaning” of the data. For this reason, the creation of instant memos during or immediately after the episode is highly valuable. These memos may take the form of short notes or keywords that capture elements judged relevant to the study aim. Their importance lies in the fact that the researcher is never neutral during the episode but always present as an analytic vector. With the partial exception of the first episode, each encounter is also informed by the analytic work already completed. This occurs formally, for example through changes to an interview guide, and practically, through the researcher’s emerging conceptual understanding of earlier data. As a result, when themes recur in subsequent episodes, they are never encountered in a wholly unstructured way. They are already being approached through partial analytic development, even if that development remains provisional. New nuances may still emerge, and meanings may shift, diverge, or consolidate. Especially in the early stages, it is often difficult to anticipate where these meanings will lead. This is precisely why instant memos are so important: they preserve early analytic movement and accompany recordings or field notes with initial conceptual development.
Researcher debriefing	This may take different forms, such as journalling or voice notes, but in all cases, it provides an opportunity to record reactions, doubts, methodological concerns, and emotionally salient aspects of the encounter. Debriefing captures concepts that arise directly from the episode while the experience is still close. It may also document perceived limitations, field-based uncertainties, or sources of distress relevant to the research process. For this reason, debriefing is most useful when it takes place soon after the episode, once the researcher has left the field or the interview context.
Transcript	A further step is the production of the transcript or, more broadly, the preparation of the unit of analysis that will later be formally coded. Although this stage may appear mainly technical, it is analytically delicate. A high-quality transcript allows the integration of what has occurred up to that point, including the episode itself (i.e. the verbatim transcription of participants’ accounts, notes on non-verbal communication, and observations about participants’ emotional states), instant memos, and debriefing. In this way, it preserves richness of meaning and brings together different levels of early analysis.

**Fig. 1:**
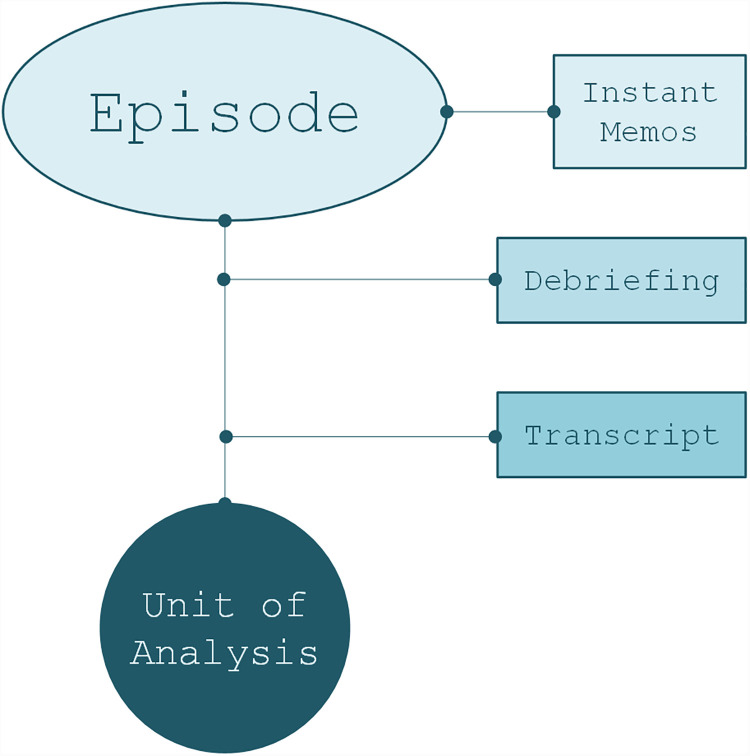
Phases of the preliminary meaning creation.

Fig. 1 presents a graphical representation of these phases, reflecting their sequential and logical progression within the process. Importantly, preliminary meaning creation is not a single event but a recurring sequence that is repeated across all episodes included in the study.

**Fig. 2:**
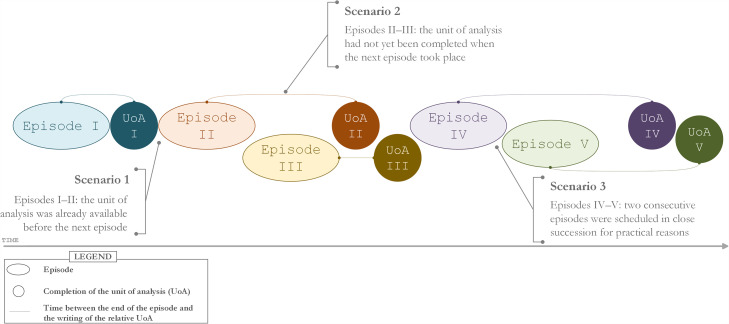
Timeline illustrating three hypothetical scenarios between fieldwork episodes and their corresponding units of analysis.

Regarding the organization of the episodes, one practical aspect should be highlighted. Research settings are often complex, and the organisation of episodes may require considerable flexibility. Fig. 2 further illustrates this aspect by showing a hypothetical “process diagram” that includes several scenarios researchers may encounter in practice. At times, a unit of analysis may already be available between one episode and the next (episodes I–II in Fig. 2). In other cases, the unit of analysis from the previous episode may not yet have been completed before the next episode takes place (episodes II–III in Fig. 2). It may also happen that two consecutive episodes need to be scheduled very close together for practical reasons (episodes IV–V in Fig. 2). These scenarios can vary widely. What remains constant, however, is the need to preserve the quality of each unit of analysis. This depends not only on the time available between the episode and transcription, but also, and more importantly, on the quality of instant memos and debriefing and on their conscious integration into the analytic process. For this reason, the organisation of research activities should, as far as possible, ensure that each episode is accompanied by its own preliminary meaning creation phase.

The second group of activities is the *meaning integration phase*. Whereas the preliminary meaning creation phase is centred on a more defined set of activities leading from each research episode to the corresponding unit of analysis, this phase involves increasing analytic complexity. With each new unit of analysis, the work of comparison and integration expands, requiring meanings to be progressively refined and connected within a broader analytic account.

Table 3 details the activities encompassed in the *Meaning integration phase* and their description.

**Table 3:** Activities of the meaning intergation phase and their description.

Activity	Description
Coding	Coding is the phase in which units of analysis are examined and assigned analytic labels. More broadly, it involves comparison across data segments to move towards increasingly abstract concepts. Rather than functioning as a mechanical hierarchy of broader and broader groupings, coding works as an integrative process that refines meanings and clarifies their complexity and interrelationships [23]. Across GT traditions, this phase is described using different terms, such as substantive, open, initial, axial, or focused coding, but all of these refer to a process that begins with close engagement with individual units of analysis and gradually relates these units to one another. Through this process, broader patterns, themes, or categories begin to emerge. Although this movement can be described analytically as if it unfolds in stages, in practice it is continuous. Coding one unit of analysis inevitably influences the coding of subsequent units, and analytic understanding progressively expands as the researcher moves through the data. In this sense, overarching analysis is not something added only at a later stage; it is gradually constructed throughout coding. Because of this, coding should follow reasonably soon after the unit of analysis is prepared, so that developing insights can inform decisions about subsequent episodes.
Analytical memos	Analytic memos are a further key element of this phase. They may include keywords, labels, conceptual links, or short reflections that capture the development of meanings as analysis progresses. They may confirm earlier instant memos, extend them conceptually, contribute to the naming of themes or categories, or generate further chains of analytic reflection. Memos therefore serve both semantic and organisational functions. Their use is highly personal and can be implemented in many ways. However, within the complexity of GT, memo writing is a fundamental practice for supporting conceptual organisation and analytic development.
Analytical consolidation	Analytic consolidation refers to the point at which identified meanings are brought together into a broader matrix of interpretation. At this stage, individual units of meaning, whether expressed as categories, themes, or conceptual elements, are related to one another in a more coherent whole. This requires cyclical analysis. The process involves not only comparison across units of analysis, but also continuous questioning of categories, themes, memos, and interpretations until they make sense both in relation to the research question and in relation to one another. From this perspective, even the earliest data are repeatedly revisited. Although the first episode may initially appear to have a more superficial analytic level, the cyclical nature of GT means that it is later reinterpreted considering the broader analytic development. Early observations can therefore be refined, reworked, or repositioned as the overall account becomes more conceptually developed. A practical and common example of this process is the realisation that a theme initially identified as independent is, in terms of its specific meaning, better understood as a subtheme of a broader theme. Conversely, what is initially identified as a subtheme may progressively gain interpretive autonomy and become meaningful enough to be treated as a theme in its own right.
Graphical representation	Because GT findings are often complex, graphical representation can play an important role in the final stages of analysis. Visual representation does not merely summarise results. It can also clarify the components of the analytic account, how these components are connected, and how they influence one another.

**Fig. 3:**
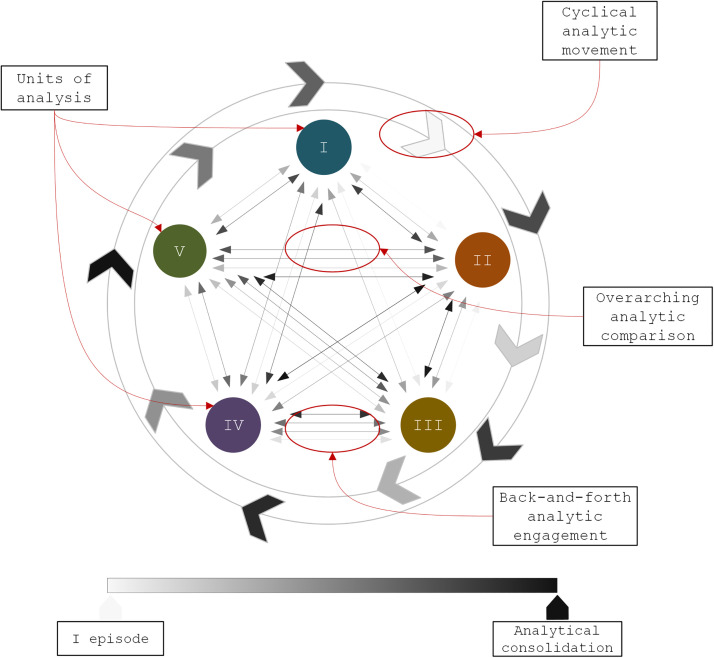
Meaning integration phase.

Fig. 3 illustrates the complexity of the meaning integration phase by representing its main analytic directions, namely the cyclical movement, the overarching comparison, and the back-and-forth engagement across data and concepts. The figure should be understood only as an example of the process rather than as a fixed model. Every GT study is unique in relation to its data, focus, and researchers, and the analytic movement is therefore also unique. A key principle of GT is that the data drive the analytic movement and indicate its direction.

Taken together, these phases highlight the centrality of the researcher in GT. The researcher is not only the interpretive lens through which the data are read, interpreted, and analysed, but also the point at which the development of meaning is retained and progressively integrated. Across the whole process, the researcher remains in continuous relation with the data and is actively involved in the creation, testing, refinement, and consolidation of meanings. Until analytic consolidation is reached, both the researcher and the analysis remain in motion. The more able the researcher is to remain flexible while also recognising and developing raw meaning, the greater the potential depth and quality of the final account. For this reason, the GT analytic process is best understood as unique, coherent, integrated, and time sensitive. Recognising and respecting these characteristics is essential for producing high-quality and methodologically sound findings.

## Illustration of data management and analysis

### Data handling as key for success

Managing and working with data in a GT study can be materially and practically demanding, especially when the research process generates large volumes of text. Data sources vary in density, and some units of analysis may be particularly rich in meaning, producing several pages in which almost every segment appears analytically relevant. Alongside the interpretive work required to analyse such material, the researcher must also read and re-read each unit of analysis to become familiar with the data and support deeper analysis. In this sense, what is often called “analysis” also includes the practical work of handling, organising, and repeatedly engaging with conceptually rich documents that must be continuously related to one another.

In the present study, particular care was devoted to the fidelity of this initial stage of data handling, starting with the interviews. Interviews were transcribed verbatim, incorporating all verbal exchanges recorded during the encounters. Non-verbal communication was also incorporated into the transcripts: an observer accompanied the researcher throughout each interview and systematically noted non-verbal cues, which were then integrated into the corresponding transcript. Paralinguistic features judged analytically meaningful, including pauses, tone of voice, and instances of irony in specific passages, were annotated directly within the text rather than treated as incidental to the spoken content. Once transcription was complete, participants were recontacted and the resulting transcripts were shared with them, so that they could confirm their satisfaction with the quality and content of what had been recorded. This attention to transcription accuracy and completeness was a necessary precondition for the subsequent, equally demanding work of organising and repeatedly engaging with the material.

For this reason, it is useful to describe how data were managed in concrete terms in the present GT study. Analysis was conducted entirely manually, using only Microsoft Word and Microsoft Excel, while drawing inspiration from approaches described in the qualitative literature and adapting them to the analytic needs of the study [24]. Transcription of the units of analysis was managed in Word documents that also contained contemporaneous instant memos and the outcomes of researcher debriefings, both recorded through the comment function. The main body of each document therefore consisted of transcribed text, while early analytic meanings were captured through embedded comments.

Initial analysis was also conducted in Word. Early coding began within the same documents, and analytic memos generated during this phase were added as comments. When an instant memo required further conceptual development, this was expanded by replying to the original comment and adding the necessary detail. When new analytic insights emerged, additional comments were created. Still within Word, and again through the comment function, preliminary hypotheses for category labels and subthemes were noted. This represented an initial and provisional step towards what might later become the final analytic structure.

Excel was introduced at a later stage to support more explicit integration across units of analysis. Only selected quotations were transferred, with careful attention to preserving traceability to their source. Each unit of analysis carried a unique identifier together with contextual information, mainly the duration of observations or interviews, the setting, and the activity observed. These identifiers were retained when quotations were extracted, so that each excerpt could always be traced back to its original source. Within Excel, quotations were grouped according to shared meaning, allowing progressively more complex and integrated meanings to be developed across multiple units of analysis.

In this study, part of the analytic structure became visible relatively early, and subsequent work mainly involved refinement and enrichment. In another area, however, an early structure that initially seemed plausible was not supported by the data during later stages of refinement and consolidation. This observation emerged from working closely with the data. In some cases, the proposed structure was not adequately supported by the data, as the relevant excerpts did not sufficiently represent the hypothesised themes. In other cases, the structure did not adequately account for the data, as some excerpts considered meaningful for the interpretation of the findings could not be placed within the proposed thematic structure. It therefore became necessary to step back, dismantle that provisional structure, and reorganise the material differently based on the data relevant to that analytic area. This revision led to a modification of the thematic structure near the end of the analytic phase, and that structural change itself enabled analytic consolidation.

This account of how units of analysis were managed is offered as a practical example that may support other researchers when planning data management in GT studies. At the same time, these phases remain highly subjective and are shaped not only by the researcher’s analytic stance, but also by the specific characteristics of the study, including its focus, topic, and data. For this reason, it is difficult to predict in advance the analytic timeline, the eventual number of categories and subthemes, or the turning points that may emerge during analysis. What remains essential is a clear understanding of the methodological premises and of the study aim, together with a willingness to set aside preconceived expectations about what the results should be, while recognising that some initial orientation inevitably derives from disciplinary knowledge and prior research experience.

Equally important is the willingness to step back when necessary and to question findings that may initially appear established. This requires trust in the analytic process. In this regard, analytic quality should not be sacrificed for speed or premature closure. Sustained analytic engagement and methodological rigour are more likely to support credible and high-quality findings, provided that the research question is coherent with GT and that data collection and analysis are conducted in line with GT principles.

A final practical point concerns software. A range of software packages is available to support qualitative analysis. Without dismissing these tools, their use requires caution and reflexive awareness, as software may encourage excessive automation of analytic activity [5]. Because GT analysis depends fundamentally on the researcher’s interpretive work, delegating too much to automated functions may weaken analytic engagement or subtly shape how the data are read. The key issue, then, is not whether software is used, but how it is used: transparently, consciously, and in ways that support rather than replace the researcher’s analytic labour [25].

### The value of time

A final methodological consideration concerns time. In GT, time is not a marginal logistical issue but a central methodological and practical constraint that should be addressed at the design stage [5]. As the previous sections suggest, the time required to conduct a GT study is closely linked to the researcher’s sustained relationship with the data throughout the analytic process.

Although this also applies, to some extent, to other qualitative approaches, in GT it has two particularly important implications: continuity of analytic engagement and the limits of automation. First, continuity refers to the extent to which the same researcher, or a small analytic team, remains actively involved in the project and closely connected to the evolving analysis over time. Given the cumulative nature of GT, sustained engagement supports analytic sensitivity, coherence, and the progressive integration of meanings, and can therefore reasonably be associated with higher-quality findings. In practical terms, this often means that the researcher involved in the first episode should remain actively engaged until analytic consolidation is achieved.

This requirement is inherently time intensive. GT benefits from close temporal proximity between data collection and analysis, and from repeated contact with the data as analysis develops. It also means that organisational strategies designed mainly to accelerate the process, such as fragmenting the work across many contributors, may introduce methodological risks. When participation is partial or intermittent, it becomes harder to develop a sufficiently comprehensive understanding of the analytic trajectory and of the decisions through which categories and interpretations have been refined. Even when handovers are carefully managed, those joining the process later have not undertaken the interpretive work that produced earlier analytic moves, and this may weaken depth and internal coherence.

At the same time, research contexts are often complex, and practical contingencies may require flexibility, including redistribution of tasks or changes in personnel. These situations need to be addressed pragmatically and ethically, balancing methodological ideals with responsibilities towards participants and with the realities of research work. The key point is that understanding why continuity matters in GT allows teams to make context-sensitive decisions while preserving, as far as possible, methodological rigour and the pursuit of high-quality findings.

Second, the central role of the researcher in GT also clarifies the limits of automation as a time-saving strategy. As discussed above, GT analysis depends on a strong interdependence between researcher and data. High-quality findings rely on the researcher’s active work in handling data, identifying meanings, developing concepts, and integrating insights across units of analysis. Because analytic potential is embedded in repeated engagement with the data, software is not successful in replacing this interpretive labour in any substantial way [5]. For this reason, the use of software is unlikely to produce a meaningful reduction in the time needed to reach analytic consolidation if analytic depth and coherence are to be preserved. Attempts to automate analytic steps may lead, even unintentionally, to the skipping of interpretive work or subtly shape what is noticed and how meanings are organised, with possible consequences for nuance and conceptual development. The relevant methodological question is therefore not simply whether software is used or avoided, but which tasks are delegated to it, how this delegation is monitored, and how sustained, reflexive engagement with the data is preserved [25]. This does not argue against software use. Rather, it highlights that choices about tools and modes of use have methodological consequences and should therefore be made deliberately, with competence and reflexive awareness.

To make these considerations more concrete, the temporal organisation of the present GT can be reported as an illustrative example. The first episode took place in July 2025, and analytic consolidation was reached in the first days of December 2025. Although the GT did not monopolise the researchers’ workload during this period, engagement with the project remained continuous across several months, spanning both preliminary meaning creation and meaning integration. Because overall calendar duration can remain partly generic when researchers are also involved in other work, a more informative indicator of practical workload is the net time devoted to the episodes themselves. The cumulative time spent conducting interviews was 6 h and 23 min, with a mean duration of 32 min per interview. Participant observation accounted for 10 h and 28 min in total, with a mean duration of 1 hour and 30 min per observation.

These figures reflect the specific characteristics of the study, including its context and participants, and should not be treated as benchmarks. However, reporting episode-level duration offers a more tangible sense of the workload involved in GT and may support more realistic planning in future projects, including the allocation of resources, the anticipation of analytic timelines, and the practical feasibility of conducting GT with methodological rigour.

## Practical implications

Beyond the specific empirical findings of the GT from which it originates, this paper is intended as a practice-oriented contribution to methodological discussion within midwifery research. Reflections of this kind, centred on the concrete decisions researchers face when applying GT rather than on restating its theoretical foundations, may help other researchers approach similar methodological junctures already somewhat prepared, for instance when negotiating the alignment between philosophical positioning and analytic practice, or when judging theoretical sufficiency within the constraints of applied healthcare research. At the same time, this paper does not claim to offer a single correct way of proceeding. It is offered as one contribution among several, in the hope that different ways of addressing shared methodological challenges can, over time, be compared and discussed within the field. Exchanges of this kind may gradually contribute to a shared, practice-oriented body of methodological knowledge in midwifery research, one that other researchers can draw upon, question, and build on further. In this sense, the practical value of this paper lies less in providing definitive answers than in taking part in an ongoing conversation that, we hope, may leave midwifery researchers feeling somewhat more empowered and motivated to engage in qualitative research, and more genuinely committed to the quality of their methodological and research choices.

## Limitations

This section refers to the limitations of the present methodological paper, rather than to the limitations of GT as a methodology or to the substantive limitations of the empirical GT study from which these reflections were developed. The main limitation is that the paper is grounded in a single GT project conducted in a specific disciplinary, professional, and national context. For this reason, the transferability of the reflections to other healthcare settings, countries, disciplines, or GT traditions should be assessed critically rather than assumed.

The account is also situated. The decision points discussed were selected because they were particularly salient in the authors’ experience, and the authors’ professional and disciplinary positioning shaped how these issues were identified and interpreted. Finally, the paper does not offer a prescriptive or formally evaluated template. Rather, it provides practice-oriented reflections intended to support methodological awareness and reflexivity in applied GT research.

## Ethics statements

The study informing this paper was conducted in accordance with the Declaration of Helsinki and was approved by the University Research Ethics Committee (CERA; approval no 2025/57) of the University of Genoa. All participants were informed about the study and provided written informed consent prior to participation.

## Funding

This research did not receive any specific grant from funding agencies in the public, commercial, or not-for-profit sectors.

## CRediT author statement

**MB:** Conceptualization, Investigation, Writing - Original Draft **FT:** Conceptualization, Writing - Review & Editing, Supervision **MZ:** Supervision **LS:** Supervision **AB:** Conceptualization, Supervision **GC:** Conceptualization, Writing - Review & Editing, Supervision.

## Declaration of competing interest

The authors declare that they have no known competing financial interests or personal relationships that could have appeared to influence the work reported in this paper.

## Data Availability

No data was used for the research described in the article.

## References

[1] Downe S., Agius J.C., Balaam M.-C., Frith L. (2020). Understanding childbirth as a complex salutogenic phenomenon: the EU COST BIRTH Action Special Collection. PLoS. One.

[2] Vermeulen J., Luyben A., O’Connell R., O’Connell M., Vivilaki V., McNeill L., Sinclair M., Fleming V., Fobelets M. (2026). Expectations, experiences and contexts of European midwives pursuing a doctoral degree: a twenty-three-country exploratory survey. J. Adv. Nurs..

[3] Brennan H.M., Furber C. (2026). Understanding the barriers UK midwives face in leading research: a critical discussion paper. Midwifery..

[4] Skeide A. (2026). Midwifery Care studies: a reflexive methodology for a practice-based science. Birth.

[5] Timonen V., Foley G., Conlon C. (2018). Challenges when using grounded theory: a pragmatic introduction to doing GT research. Int. J. Qual. Methods.

[6] Charmaz K. (2006).

[7] Corbin J., Strauss A. (2015). Techniques and Procedures for Developing Grounded Theory.

[8] Glaser B., Strauss A. (1967).

[9] Charmaz K., Thornberg R. (2021). The pursuit of quality in grounded theory. Qual. Res. Psychol..

[10] Connor J., Flenady T., Dwyer T., Massey D. (2026). The application of classic grounded theory in Nursing studies: a qualitative systematic review. J. Adv. Nurs..

[11] Sbaraini A., Carter S.M., Evans R.W., Blinkhorn A. (2011). How to do a grounded theory study: a worked example of a study of dental practices. BMC. Med. Res. Methodol..

[12] Berthelsen C.B., Grimshaw-Aagaard S., Hansen C. (2018). Developing a guideline for reporting and evaluating grounded theory research studies (GUREGT) (Pt. 1). Int. J. Health Sci..

[13] Kenny M., Fourie R. (2015). Contrasting classic, Straussian, and Constructivist grounded theory: methodological and philosophical conflicts. Qual. Rep..

[14] Mykhalovskiy E., Eakin J., Beagan B., Beausoleil N., Gibson B.E., Macdonald M.E., Rock M.J. (2018). Beyond bare bones: critical, theoretically engaged qualitative research in public health. Can. J. Public Health = Rev. Can. Santé Publique.

[15] Clarke, V., & Braun, V. (2013). *Successful qualitative research: a practical guide for beginners*.

[16] Mertens D.M. (2010). Transformative Mixed Methods Research. Qual. Inq..

[17] Heron J., Reason P. (1997). A participatory inquiry paradigm. Qual. Inq..

[18] Broom A., Willis E. (2007).

[19] Doody S., Plaisance K.S. (2025). What is ‘good’ Science? How disciplinary norms and expectations discourage broad interdisciplinary collaboration. Perspect. Sci..

[20] Corrigan P.W., Twiss M. (2026). What are the implications of investigator positionality for mental health services research?. Br. J. Psychiatry.

[21] Braun V., Clarke V. (2025). Reporting guidelines for qualitative research: a values-based approach. Qual. Res. Psychol..

[22] Braun V., Clarke V. (2021). One size fits all? What counts as quality practice in (reflexive) thematic analysis?. Qual. Res. Psychol..

[23] Eakin J.M., Gladstone B. (2020). Value-adding” analysis: doing more with qualitative data. Int. J. Qual. Methods.

[24] Macdonald M.E., Siedlikowski S., Liu K., Carnevale F.A. (2023). Introducing SAMMSA, a five-step method for producing ‘quality’ Qualitative analysis. Qual. Health Res..

[25] Jenkins N., Monaghan K., Smith M. (2023). Did they really say that? An agential realist approach to using computer assisted transcription software in qualitative data analysis. Int. J. Soc. Res. Methodol..

